# Population-specific differences in ClinVar pathogenic and likely pathogenic variants of epilepsy-associated genes between East Asian and non-finnish European populations: a nucleotide-resolved gnomAD v4.1.1 analysis

**DOI:** 10.3389/fgene.2026.1973684

**Published:** 2026-10-23

**Authors:** Jin-Xing Lai, Ming-Feng He, Zhi-Cong Fan, Fu-Jiang Yan, Bao-Zhu Guan, Xiang-Hong Liu

**Affiliations:** 1 Key Laboratory of Neurogenetics and Channelopathies of Guangdong Province and the Ministry of Education of China, Department of Neurology, Institute of Neuroscience, The Second Affiliated Hospital, Guangzhou Medical University, Guangzhou, China; 2 Department of Neurology, Ganzhou Hospital-Nanfang Hospital, Southern Medical University (Ganzhou People’s Hospital), Ganzhou, China

**Keywords:** allele ambiguity, carrier screening, ClinVar, epilepsy, gnomAD, population frequency, variant interpretation

## Abstract

Pathogenic and likely pathogenic (P/LP) variants in ClinVar guide variant interpretation in clinical genetic testing, yet their population-specific frequency differences have not been systematically examined in epilepsy. We compiled 199 epilepsy-associated genes, extracted 46,746 unique P/LP variants from ClinVar, and retrieved allele frequencies from gnomAD v4.1.1 gene-level tables using nucleotide-resolved matching. Fisher’s exact test with Benjamini-Hochberg correction (q < 0.05) identified nine variants significantly enriched in East Asian (EAS) relative to non-Finnish European (NFE) populations, with odds ratios of 36.3–867.9. Eight additional low-frequency EAS signals met the same enrichment criterion with at least two EAS alleles and an uncorrected p < 0.01 and are reported as exploratory. Thirteen of the seventeen variants reside in seven autosomal recessive genes, namely, *ALDH7A1*, *ST3GAL5*, *GOSR2*, *AARS1*, *BRAT1*, *WWOX*, and *UBA5*. In parallel, an rsID-level workflow, the standard approach in database mining, yielded 60 candidate EAS-enriched variants. Nucleotide-resolved matching confirmed only fifteen of these and additionally recovered two signals the rsID workflow missed, a false-positive rate of 75.0%. These findings show that epilepsy P/LP variant frequencies differ substantially between EAS and NFE populations and concentrate in recessive genes with direct relevance to carrier screening. rsID-level ClinVar-gnomAD integration systematically overestimates population specificity, and nucleotide-resolved allele matching should become a standard step in population frequency analyses.

## 1 Introduction

Epilepsy affects approximately 50 million people worldwide and is among the most common neurological disorders (Beghi, 2020). A substantial proportion of early-onset epilepsy has a monogenic basis. Diagnostic yields of exome sequencing and multigene panels reach 24% and 19%, respectively, in unselected cohorts (Sánchez Fernández et al., 2019; Sheidley et al., 2022). The identification of a pathogenic variant in an epilepsy gene can guide treatment selection, inform prognosis, and enable genetic counseling (Thomas and Berkovic, 2014). Clinical laboratories apply the ACMG standards for next-generation sequencing when performing this testing (Rehm et al., 2013). Population allele frequency is a cornerstone of this interpretation, because variants common in reference populations cannot cause fully penetrant, severe early-onset disease. The ACMG/AMP guidelines accordingly assign benign evidence to variants exceeding defined frequency thresholds (Richards et al., 2015; Ghosh et al., 2018).

ClinVar is the primary public archive of variant classifications (Landrum et al., 2018), and gnomAD provides allele frequencies across eight populations (Karczewski et al., 2020; Chen et al., 2024), now spanning nearly one million individuals (Sun et al., 2024). Database mining studies increasingly combine these resources at the rsID level through programmatic tools (Xin et al., 2016). This approach has characterized population-specific pathogenic variant frequencies in monogenic Parkinson disease (Flores-Ocampo et al., 2025), cardiomyopathy (Rosamilia et al., 2024), and Brugada syndrome (Chen et al., 2018). A single dbSNP identifier can nevertheless aggregate several distinct nucleotide changes at one position. Programmatic rsID lookups typically return the frequency of the most common allele without specifying which nucleotide change it represents. The extent to which this conflation distorts population enrichment analyses has not been quantified.

For epilepsy, systematic characterization of population-specific differences in P/LP variant frequencies has not been performed, despite epilepsy genetic testing being among the most commonly ordered worldwide. A recent comprehensive classification identified 168 genes in which epilepsy is the core or pure symptom (Zhang et al., 2024), and the catalogue of epilepsy-associated genes continues to expand through cohort and literature studies (Gu et al., 2024). Approximately 5.7% of ClinVar variants have conflicting interpretations, and 78% of clinically relevant genes harbor at least one discordant variant (Lazareva et al., 2024). These observations, together with the known ancestry bias in genomic databases (Ramaswamy et al., 2022; Sharo et al., 2023; Venner et al., 2024) and its extension to admixed and South Asian populations (Bianco and Planello, 2025; Rony et al., 2026), motivated our analysis. We assembled 199 epilepsy-associated genes and compared EAS and NFE allele frequencies of ClinVar P/LP variants using nucleotide-resolved matching against gnomAD v4.1.1 gene-level tables. We additionally quantified the false-positive rate of the standard rsID-level workflow.

## 2 Materials and methods

### 2.1 Gene selection

We assembled 199 epilepsy-associated genes. The core set comprised 168 genes in which epilepsy is the pure or core symptom, as defined by Zhang et al. (2024). This classification integrates ClinGen Epilepsy Gene Curation Expert Panel assessments, PanelApp Epilepsy panel designations, and OMIM annotations. We added 31 neurodevelopmental disorder genes that are routinely included in clinical epilepsy gene testing and that share molecular pathways with core epilepsy genes. The source of each gene (core epilepsy or NDD expansion) is annotated in Supplementary Table S1.

### 2.2 ClinVar data retrieval and processing

We downloaded the ClinVar variant summary file (variant_summary.txt.gz; ClinVar monthly release dated 1 July 2026) from the NCBI FTP server (https://ftp.ncbi.nlm.nih.gov/pub/clinvar/variant_summary.txt.gz) on 1 July 2026. We extracted records for the 199 target genes with clinical significance of Pathogenic or Likely pathogenic. After deduplication by chromosome, position, reference allele, and alternate allele, 46,746 unique P/LP variants remained across 191 genes. Among these, 23,316 single nucleotide variants corresponded to 9,847 unique rsIDs. ClinVar and gnomAD v4.1.1 both use the GRCh38 reference genome, so no liftover was required; variants were normalized to the chromosome:position:reference:alternate format.

Variant identity was defined throughout by full GRCh38 coordinates (chromosome, position, reference allele, and alternate allele). Protein-level annotations were used only for reporting. All deduplication and all matching between ClinVar and gnomAD were ultimately resolved at the nucleotide level, because distinct nucleotide substitutions can produce the same amino acid change.

Variants were selected using the aggregate germline classification recorded in the ClinicalSignificance field of the ClinVar variant summary file. Records whose aggregate classification contained “Pathogenic” or “Likely pathogenic” were retained, and records annotated as “Uncertain significance”, “Benign”, “Likely benign”, “drug response”, or “risk factor” were excluded. Review status and the number of submitters were retained for each variant, and no independent ACMG/AMP re-curation was performed.

### 2.3 Nucleotide-resolved gnomAD v4.1.1 frequency retrieval

We retrieved allele frequencies from the gnomAD v4.1.1 gene-level variant tables, which provide population-specific allele counts and allele numbers aggregated across gnomAD exome and genome sequencing on the GRCh38 reference genome. For each P/LP SNV we aligned the ClinVar c. and p. notation, expressed on a RefSeq (NM_) transcript, to the HGVS and protein consequences of the allele at the same rsID in the gnomAD table. Protein notation was matched first, because it is robust to transcript differences for missense and nonsense variants; this protein-level match was then confirmed against the genomic coordinates, reference allele, and alternate allele recorded in the gnomAD gene-level table, so that final identity was always established at the nucleotide level. The c. notation was matched for splice variants, which lack protein notation. When c. numbering differed across transcripts, splice variants were matched by consequence class, requiring a single splice-region allele at the position. Splice-region and start-loss variants were then verified manually against the gnomAD genomic coordinates, reference allele, and alternate allele. Among the 2,160 rsIDs with gnomAD data, 805 yielded an informative nucleotide-resolved match; at the remaining positions the ClinVar P/LP allele was absent from gnomAD or only a different allele carried frequency data. A flowchart of the matching procedure is provided as Supplementary Figure S1, and the complete study workflow is shown in Figure 1. Population-specific allele counts and allele numbers were extracted for EAS, NFE, South Asian (SAS), African/African American (AFR), and Admixed American (AMR) populations.

**FIGURE 1:**
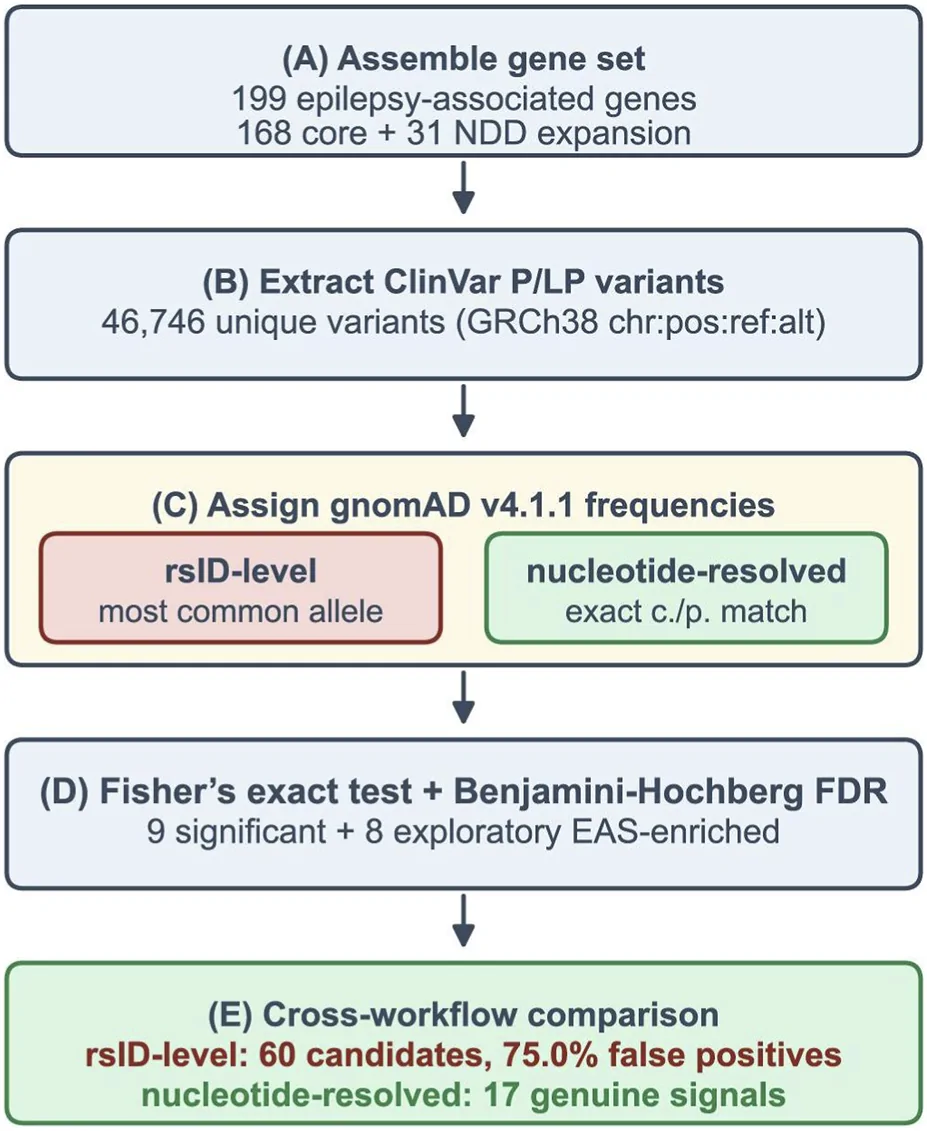
Study workflow. **(A)** Assembly of the 199 epilepsy-associated genes (168 core epilepsy genes plus 31 neurodevelopmental disorder expansion genes). **(B)** Extraction of ClinVar pathogenic/likely pathogenic records and deduplication by GRCh38 chromosome, position, reference allele, and alternate allele. **(C)** Assignment of gnomAD v4.1.1 population frequencies through two parallel channels: an rsID-level channel (frequency of the most common allele, mirroring programmatic lookups) and a nucleotide-resolved channel (exact c./p. matching to the specific allele). **(D)** Fisher’s exact testing with Benjamini-Hochberg FDR correction, identifying nine significant and eight exploratory EAS-enriched variants. **(E)** Cross-workflow comparison quantifying the false-positive rate of the rsID-level channel.

Gene-level gnomAD v4.1.1 variant tables were exported from the gnomAD browser (https://gnomad.broadinstitute.org/).

### 2.4 rsID-level workflow for methodological comparison

To quantify the impact of allele conflation, we repeated the analysis with a standard rsID-level workflow. For each rsID, we assigned the frequency of the most common allele, aggregated across exome and genome data, without verifying which nucleotide change it represented. This workflow mirrors programmatic rsID-based lookups implemented by annotation resources (Xin et al., 2016). The two workflows used identical statistical tests, allowing a direct comparison of candidate sets.

### 2.5 Statistical analysis

For each P/LP variant with a nucleotide-resolved EAS or NFE allele number, we constructed a 2 × 2 contingency table from the population-specific allele counts and allele numbers and applied Fisher’s exact test. The significance and exploratory layers tested the same 805 informative variants, each with its own allele numbers. Significance was defined as a Benjamini-Hochberg-corrected q value below 0.05 together with EAS enrichment (NFE frequency of zero, or EAS frequency at least twofold higher than NFE). Exploratory low-frequency signals were defined as nucleotide-confirmed variants that did not reach q < 0.05 but had at least two EAS alleles and an uncorrected Fisher p value below 0.01. They additionally had to meet the same EAS enrichment criterion. The threshold of two EAS alleles was chosen because a single EAS allele cannot be distinguished from a sequencing error and cannot reach p < 0.01 even when the variant is absent from NFE. Because the exploratory threshold was not corrected for the 805 tests, these signals are presented separately and interpreted cautiously. With EAS allele numbers of about 44,900 and NFE allele numbers of about 1,180,000, a variant absent from NFE requires at least two EAS alleles to reach p < 0.01. A single EAS allele yields p = 3.7 × 10^−2^. The analysis is therefore insensitive to EAS-specific enrichment below an allele frequency of about 4.5 × 10^−5^ (Supplementary Figure S2). We estimated effect sizes as odds ratios (OR) with Woolf 95% confidence intervals (CI) using the Haldane-Anscombe correction, which adds 0.5 to each cell of the 2 × 2 table.

All analyses were performed in Python 3.12 using SciPy (Fisher’s exact test via scipy. stats.fisher_exact) and statsmodels (Benjamini-Hochberg correction via statsmodels. stats.multitest). The complete analysis code is available from the corresponding author upon reasonable request and will be deposited in a public repository upon acceptance.

### 2.6 Gene constraint metrics

Gene-level constraint metrics were obtained from gnomAD (Karczewski et al., 2020; Chen et al., 2024), including the probability of loss-of-function intolerance (pLI), missense Z-score, and LOEUF. Constraint data were available for all 199 genes.

## 3 Results

### 3.1 Dataset characteristics

The final gene set comprised 199 epilepsy-associated genes (Table 1): 168 core epilepsy genes and 31 neurodevelopmental disorder expansion genes. ClinVar contained 46,746 unique P/LP variants across 191 genes, of which 23,316 were single nucleotide variants (SNVs) corresponding to 9,847 unique rsIDs. Eight genes carried no P/LP variants (*ICK*, *March 6*, *PIGP*, *RAPGEF2*, *SLC7A6OS*, *TNRC6A*, *UGP2*, and *YEATS2*). The genes with the largest P/LP burdens were *NF1* (7,461), *SCN1A* (3,129), and *TSC2* (2,355). Gene-level constraint analysis confirmed strong loss-of-function intolerance across the set: 104 of 199 genes (52.3%) had pLI above 0.9, 78 (39.2%) had a missense Z-score above 3, and 101 (50.8%) had LOEUF below 0.35.

**TABLE 1:** Dataset characteristics and gene constraint summary.

Category	Value
Genes analyzed, total	199
Core epilepsy genes	168
NDD expansion genes	31
ClinVar P/LP variants, unique	46,746
Genes with P/LP variants	191
Single nucleotide variants (SNVs)	23,316
Unique rsIDs	9,847
Informative variants (nucleotide-resolved, EAS or NFE AF > 0)	805
Genes with informative variants	131
Genes with constraint data	199
pLI > 0.9, n (%)	104 (52.3%)
Missense Z > 3, n (%)	78 (39.2%)
LOEUF < 0.35, n (%)	101 (50.8%)
pLI, median (IQR)	0.911 (0.007–1.000)
Missense Z, median (IQR)	2.584 (0.913–3.665)
LOEUF, median (IQR)	0.341 (0.174–0.645)

### 3.2 Frequency distribution of nucleotide-resolved P/LP variants

Nucleotide-resolved matching against gnomAD v4.1.1 gene-level tables assigned allele frequencies to 805 informative P/LP SNVs across 131 genes. The EAS and NFE distributions were markedly asymmetric. Twenty-eight variants had nonzero EAS frequency only, 720 had nonzero NFE frequency only, and 57 were present in both populations (Table 2). The predominance of NFE-only variants reflects the larger NFE sample in gnomAD and the European-ancestry ascertainment bias of ClinVar submissions. The EAS-only and shared variants, 85 in total, constituted the pool from which EAS-enriched signals were identified.

**TABLE 2:** Frequency distribution of nucleotide-resolved informative P/LP variants (EAS vs. NFE).

Category	Count
Informative variants (EAS or NFE AF > 0)	805
Nonzero in EAS only	28
Nonzero in NFE only	720
Nonzero in both populations	57
Significantly EAS-enriched (FDR q < 0.05)	9
Exploratory low-frequency EAS signals	8
Total informative variants	805

### 3.3 Nucleotide-resolved EAS-enriched P/LP variants

Nucleotide-resolved matching identified nine P/LP variants significantly enriched in EAS relative to NFE (FDR q < 0.05; Table 3). The strongest signals were the *ST3GAL5* splice donor variant c.13 + 1G > A and *ALDH7A1* p. Tyr516Cys. The *ST3GAL5* variant occurred in 28 of 41,244 EAS alleles versus 2 of 418,078 NFE alleles (q = 1.4 × 10^−24^). The *ALDH7A1* variant occurred in 16 of 44,882 EAS alleles and was absent from NFE (q = 4.3 × 10^−21^). The remaining variants comprised *GOSR2* p. Arg107Ter (12 of 44,884), *SETD1A* p. Arg913Cys (8 of 44,876), and *ALDH7A1* c.871 + 5G > A (6 of 44,882). The others were *AARS1* p. Arg840Ter (6 of 44,902), *KCNT1* p. Arg25Trp (5 of 44,816), *WWOX* c.2T > C, a start-loss variant (4 of 40,686), and *BRAT1* p. Leu59Pro (3 of 44,884). Four variants were absent from NFE; the other five showed EAS allele frequencies 38- to 158-fold higher than their NFE frequencies. Odds ratios ranged from 36.3 (*WWOX* c.2T > C) to 867.9 (*ALDH7A1* p. Tyr516Cys). The nine variants mapped to eight genes and comprised six Pathogenic and three Likely pathogenic classifications. Two variants, *GOSR2* p. Arg107Ter and *BRAT1* p. Leu59Pro, carried no assertion criteria in ClinVar (zero stars), and their classifications should be reassessed.

**TABLE 3:** Nucleotide-resolved P/LP variants significantly enriched in EAS (FDR q < 0.05). Odds ratios use the Haldane-Anscombe correction with Woolf 95% confidence intervals. Stars indicate ClinVar review status (0 = no assertion criteria, 1 = single submitter, 2 = multiple submitters, no conflicts).

Gene	Inh	Transcript	Variant	EAS (AC/AN)	NFE (AC/AN)	OR (95% CI)	Stars	FDR q
*ST3GAL5*	AR	NM_003896.4	c.13 + 1G > A (splice donor)	28/41,244	2/418,078	115.6 (31.7–421.3)	1	1.4 × 10^−24^
*ALDH7A1*	AR	NM_001182.5	p.Tyr516Cys	16/44,882	0/1,180,030	867.9 (52.1–14,467.7)	2	4.3 × 10^−21^
*GOSR2*	AR	NM_004287.5	p.Arg107Ter	12/44,884	6/1,180,026	50.6 (19.6–130.5)	0	2.4 × 10^−11^
*SETD1A*	AD	NM_014712.3	p.Arg913Cys	8/44,876	3/1,179,986	63.9 (18.4–221.8)	2	9.8 × 10^−8^
*ALDH7A1*	AR	NM_001182.5	c.871 + 5G > A	6/44,882	0/1,179,884	341.8 (19.3–6,067.6)	1	3.9 × 10^−7^
*AARS1*	AR	NM_001605.3	p.Arg840Ter	6/44,902	1/1,180,038	113.9 (19.3–672.3)	1	2.2 × 10^−6^
*KCNT1*	AD	NM_020822.3	p.Arg25Trp	5/44,816	0/1,179,372	289.5 (16.0–5,235.9)	1	7.6 × 10^−6^
*WWOX*	AR	NM_016373.4	c.2T > C (start loss)	4/40,686	3/1,149,400	36.3 (9.0–146.9)	1	3.6 × 10^−3^
*BRAT1*	AR	NM_152743.4	p.Leu59Pro	3/44,884	0/1,180,034	184.0 (9.5–3,563.3)	0	3.6 × 10^−3^

### 3.4 Exploratory low-frequency signals and recessive-gene concentration

Eight additional nucleotide-confirmed variants met the exploratory criteria (EAS allele count ≥ 2, uncorrected p < 0.01, EAS enrichment), although their low allele counts precluded significance after multiple-testing correction (Table 4). These comprised *AARS1* p. Arg330Ter (3 of 44,886), *WWOX* p. Glu171Ter (2 of 44,864), and *WWOX* c.172 + 1G > C (2 of 44,874). The remaining variants were *ST3GAL5* c.662 + 2T > C (2 of 44,894), *ADGRV1* p. Trp1139Leu (2 of 44,692), and *WWOX* p. Arg54Ter (3 of 44,870). The final two variants were *UBA5* p. Arg246Ter (2 of 44,856) and *KCNC1* p. Arg164Trp (2 of 44,390). All eight showed EAS frequencies 15.8- to 52.6-fold higher than NFE or were absent from NFE, and their odds ratios ranged from 16.7 to 131.5. Two of the eight, *WWOX* p. Arg54Ter and *KCNC1* p. Arg164Trp, had raw EAS counts that did not exceed their NFE counts. Their odds ratios (16.7 and 26.5) reflect the smaller EAS reference sample and require cautious interpretation. Across the seventeen confirmed variants, thirteen resided in seven autosomal recessive genes (*ALDH7A1*, *ST3GAL5*, *GOSR2*, *AARS1*, *BRAT1*, *WWOX*, and *UBA5*), and four mapped to dominant genes (*SETD1A*, *KCNT1*, *ADGRV1*, and *KCNC1*) (Figure 2). The two *ALDH7A1* variants together accounted for 22 of 44,882 EAS alleles (0.05%), and the *ST3GAL5* splice donor variant accounted for 28 of 41,244 EAS alleles (0.07%). To define the ancestry boundary of the enrichment, we extracted SAS, AFR, and AMR frequencies for all seventeen variants (Supplementary Table S2). Fifteen variants were absent or singletons in SAS, AFR, and AMR, supporting EAS-specific or EAS-dominant enrichment. *ST3GAL5* c.13 + 1G > A remained the clearest EAS-specific signal, with an EAS frequency 52-fold higher than its SAS frequency (1 of 74,612) and absence from AFR and AMR. *WWOX* p. Arg54Ter showed a shared EAS-SAS enrichment, with comparable frequencies of 6.7 × 10^−5^ in EAS and 3.3 × 10^−5^ in SAS. This variant is best regarded as East and South Asian enriched rather than East Asian specific. Among the seventeen variants, four carried two-star ClinVar review status, ten carried one star, and three (*GOSR2* p. Arg107Ter, *BRAT1* p. Leu59Pro, and *UBA5* p. Arg246Ter) carried no assertion criteria. Restricting to variants with at least one star left fourteen signals (seven significant and seven exploratory), of which ten were recessive. Restricting to at least two stars left four signals, of which three were recessive. The recessive-gene concentration was therefore not driven by low-confidence classifications. Applying stricter exploratory thresholds did not change the qualitative conclusion: requiring at least three EAS alleles left eleven signals, and requiring an uncorrected p < 0.005 left sixteen. In both cases the recessive-gene proportion remained above 80%. As a sensitivity check, restricting the analysis to the 168 core epilepsy genes changed the signal set only by removing variants from the 31 expansion genes, and all seventeen signals mapped to core epilepsy genes.

**TABLE 4:** Exploratory low-frequency P/LP variants confirmed at the nucleotide level (EAS ≥ 2 alleles, uncorrected p < 0.01, EAS enrichment). Odds ratios use the Haldane-Anscombe correction with Woolf 95% confidence intervals. Stars indicate ClinVar review status (0 = no assertion criteria, 1 = single submitter, 2 = multiple submitters, no conflicts).

Gene	Inh	Transcript	Variant	EAS (AC/AN)	NFE (AC/AN)	OR (95% CI)	Stars	Fisher p (uncorrected)
*AARS1*	AR	NM_001605.3	p.Arg330Ter	3/44,886	3/1,180,024	26.3 (6.0–115.7)	2	9.1 × 10^−4^
*WWOX*	AR	NM_016373.4	p.Glu171Ter	2/44,864	0/1,179,866	131.5 (6.3–2,739.2)	1	1.3 × 10^−3^
*WWOX*	AR	NM_016373.4	c.172 + 1G > C (splice donor)	2/44,874	0/1,179,866	131.5 (6.3–2,738.6)	1	1.3 × 10^−3^
*ST3GAL5*	AR	NM_003896.4	c.662 + 2T > C (splice donor)	2/44,894	0/1,180,014	131.4 (6.3–2,737.7)	1	1.3 × 10^−3^
*ADGRV1*	AD	NM_032119.4	p.Trp1139Leu	2/44,692	0/1,171,262	131.0 (6.3–2,729.7)	1	1.4 × 10^−3^
*WWOX*	AR	NM_016373.4	p.Arg54Ter	3/44,870	5/1,179,600	16.7 (4.4–63.9)	2	2.4 × 10^−3^
*UBA5*	AR	NM_024818.6	p.Arg246Ter	2/44,856	1/1,179,924	43.8 (5.8–331.9)	0	3.9 × 10^−3^
*KCNC1*	AD	NM_001112741.2	p.Arg164Trp	2/44,390	2/1,175,254	26.5 (4.6–152.8)	1	7.6 × 10^−3^

**FIGURE 2:**
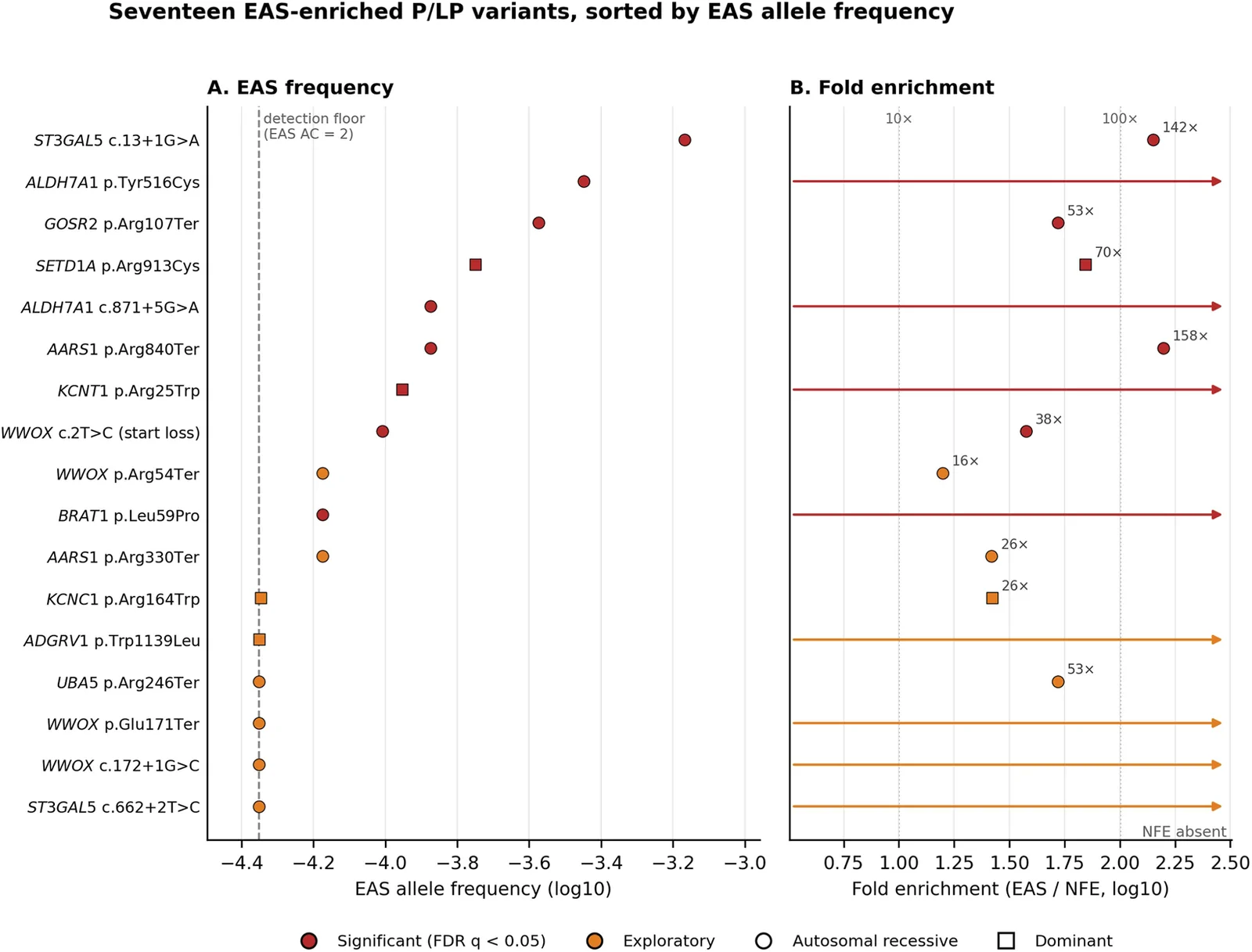
The seventeen EAS-enriched P/LP variants, sorted by EAS allele frequency. **(A)** EAS allele frequency on a log10 scale, with the detection floor (EAS AC = 2) indicated by a dashed line. **(B)** Fold enrichment (EAS/NFE) on a log10 scale. Red circles denote the nine significant variants (FDR q < 0.05) and orange circles the eight exploratory variants; circles mark autosomal recessive genes and squares mark dominant genes. Variants absent from NFE are shown as right-pointing arrows.

With respect to molecular consequence, ten of the seventeen variants (58.8%) were loss-of-function (six nonsense, three canonical splice-donor, and one start-loss), six (35.3%) were missense, and one (5.9%) was a non-canonical splice-region variant (ALDH7A1 c.871 + 5G > A). The complete consequence annotation is provided in Supplementary Table S2.

### 3.5 Methodological comparison: rsID-level versus nucleotide-resolved workflows

To quantify the methodological impact of rsID-level allele conflation, we repeated the enrichment analysis using the standard rsID-level approach. For each rsID, the frequency of the most common allele was assigned without verifying which nucleotide change it represented. This workflow yielded 60 candidate EAS-enriched variants. Nucleotide-resolved matching confirmed only fifteen of these as genuine P/LP signals, corresponding to a false-positive rate of 45 of 60 (75.0%) (Figure 3). The rsID workflow additionally missed two genuine signals, UBA5 p. Arg246Ter and KCNC1 p. Arg164Trp, that nucleotide-resolved matching recovered. Among the 45 false positives, 20 arose when a synonymous allele was the most common at the position. Another 20 arose when a distinct protein-altering allele was the most common, 4 arose from splice-region conflation, and 1 arose from a tri-allelic start-loss position (Table 5; Supplementary Table S3). In each case the ClinVar P/LP allele was a different nucleotide change at the same position. The rsID workflow assigned an informative frequency to 1,707 variants, compared with 805 under nucleotide-resolved matching. The difference reflects positions at which the ClinVar P/LP allele is absent from gnomAD but another allele at the same position carries frequency data. The full candidate-set comparison between the two workflows is provided in Supplementary Table S4. Among the 2,160 rsIDs with gnomAD data, 278 (12.9%) were multi-allelic. These results show that rsID-level integration inflates population-specific enrichment calls and that the inflation concentrates in the candidate set, because a common benign allele is precisely what marks a position as enriched.

**FIGURE 3:**
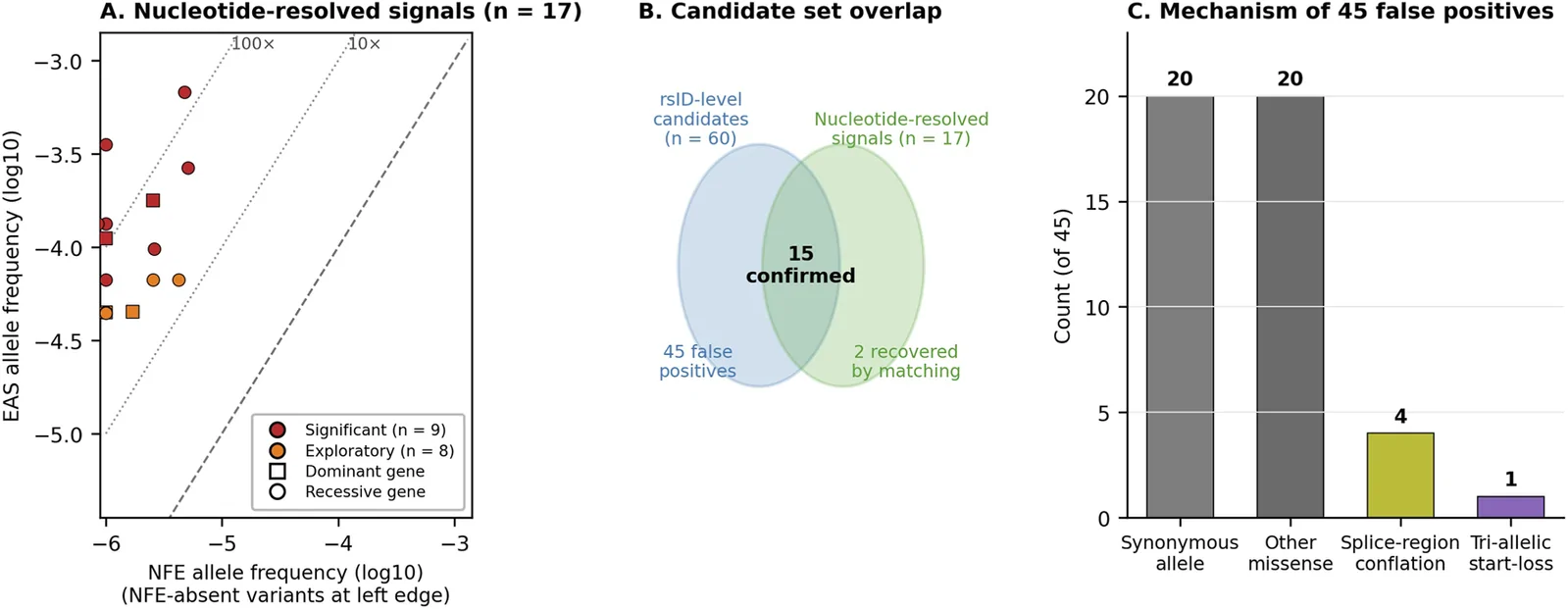
Comparison of rsID-level and nucleotide-resolved workflows. **(A)** EAS and NFE allele frequencies of the seventeen nucleotide-confirmed signals. Red circles mark the nine significant variants (FDR q < 0.05) and orange circles the eight exploratory variants; circles denote autosomal recessive genes and squares denote dominant genes. The diagonal marks equal EAS and NFE frequency, with 10-fold and 100-fold enrichment lines; variants absent from NFE are placed at the left edge. **(B)** Overlap of the 60 rsID-level candidates with the seventeen nucleotide-resolved signals: fifteen were confirmed, 45 were false positives, and two signals were recovered only by nucleotide-resolved matching. **(C)** Mechanisms of the 45 false positives.

**TABLE 5:** Mechanism of the 45 rsID-level false positives among the 60 candidates.

Mechanism	Count	Example
Synonymous allele most common	20	SZT2 p.Tyr362Tyr (not p.Tyr362Ter)
Distinct protein-altering allele most common	20	NHLRC1 p.Ser364Leu (not p.Ser364Ter)
Splice-region conflation	4	*ADGRV1* c.13434–2A>G (not A>T)
Tri-allelic start-loss conflation	1	*GOSR2* p.Met1Leu (not p.Met1?)

## 4 Discussion

Using nucleotide-resolved matching against gnomAD v4.1.1, we identified seventeen EAS-enriched P/LP variants across 199 epilepsy-associated genes. Nine were statistically significant, and thirteen of seventeen resided in seven autosomal recessive genes. In parallel, we showed that the standard rsID-level workflow inflates population-specific enrichment calls by 75.0%. These findings establish that epilepsy P/LP variant frequencies differ substantially between EAS and NFE populations and that nucleotide-resolved matching is essential for accurate population frequency analyses.

The concentration of nucleotide-confirmed signals in recessive genes has direct clinical implications. The *ALDH7A1* variants p. Tyr516Cys and c.871 + 5G > A together account for 22 of 44,882 EAS alleles, a carrier frequency of approximately 0.05%. The *ST3GAL5* splice donor variant reaches 0.07% in EAS. These values inform carrier screening and the application of PM2 and BS1 criteria in East Asian populations. The application of these frequency thresholds is being refined by gene-specific specifications, including the ClinGen Epilepsy Sodium Channel Variant Curation Expert Panel recommendations (Smith et al., 2026), and by updated frequency criteria for secondary finding genes (Johnston et al., 2026). Recessive epilepsy genes such as *ALDH7A1*, *ST3GAL5*, *GOSR2*, *AARS1*, and *UBA5* carry the bulk of this population-specific P/LP burden. Ancestry-specific carrier frequency estimation from gnomAD has been established for autosomal and X-linked recessive conditions (Hotakainen et al., 2025) and is being used to guide more equitable carrier screening (Schmitz et al., 2025). Earlier *CFTR* analyses showed that carrier frequencies in non-European populations are systematically underappreciated when European data are assumed (Nappo et al., 2020), and genetic prevalence estimation depends on the same population-resolved allele frequencies (Kurtovic-Kozaric et al., 2024). Ancestry-matched frequency data also shape the interpretation of secondary findings in multiethnic cohorts (Saeidian et al., 2024). Each of the seven recessive genes is an established cause of autosomal recessive epilepsy, including *ALDH7A1* (pyridoxine-dependent epilepsy), *ST3GAL5* (GM3 synthase deficiency), *GOSR2* (progressive myoclonus epilepsy), and *WWOX* (epileptic encephalopathy). Our study quantifies the East Asian population frequencies of their P/LP variants rather than claims their discovery. For dominant genes, the signals require cautious interpretation. The *SETD1A* p. Arg913Cys variant at 8 of 44,876 EAS alleles approaches the 0.02% threshold commonly used for the BS1 criterion and may reflect incomplete penetrance rather than a fully penetrant epilepsy allele. *KCNT1* p. Arg25Trp, *ADGRV1* p. Trp1139Leu, and *KCNC1* p. Arg164Trp were present at low frequency. Three of the seventeen variants, *GOSR2* p. Arg107Ter, *BRAT1* p. Leu59Pro, and *UBA5* p. Arg246Ter, carried no assertion criteria in ClinVar, and their classification should be reassessed. Classification reliability is a broader concern. Misclassified ClinVar variants have previously been identified through disease population prevalence (Shah et al., 2018), common pathogenic variants often undergo downgrade on reinterpretation (Xiang et al., 2020), and gene-disease associations can themselves be refuted, as demonstrated for *SCN9A* (Ghanty et al., 2025).

Among the seventeen variants, the ten loss-of-function variants (nonsense, canonical splice-donor, and start-loss) carry the strongest prior for pathogenicity under ACMG/AMP criterion PVS1 and are the highest-priority subset for re-interpretation against ancestry-matched frequency data.

Our methodological comparison quantifies a problem that is underrecognized in database mining. A single rsID can aggregate several nucleotide changes, and programmatic lookups return the frequency of the most common allele (Xin et al., 2016). We found that this produces a 75.0% false-positive rate among candidate population-specific variants. The conflation concentrates in the candidate set because a common benign allele is exactly what marks a position as enriched. This selection effect explains why our measured rate far exceeds the 12.9% multi-allelic rate among rsIDs with gnomAD data. The 902 variants that were informative only under the rsID workflow reinforce this mechanism: their positions carry frequency data in gnomAD, but the ClinVar P/LP allele itself does not. Our results extend earlier observations of ancestry bias in ClinVar (Ramaswamy et al., 2022; Sharo et al., 2023; Venner et al., 2024). They suggest that published population-specific variant lists derived from rsID-level queries, such as those in monogenic Parkinson disease (Flores-Ocampo et al., 2025) and Brugada syndrome (Chen et al., 2018), warrant nucleotide-level revalidation. Nucleotide-resolved matching by HGVS or genomic coordinates with reference and alternate alleles should become a standard step in ClinVar-gnomAD integration studies.

This study has limitations. The analysis was restricted to SNVs, and the conclusions apply to the SNV level. gnomAD v4.1.1 does not provide population-comparable frequencies for insertions, deletions, or copy-number variants, so genes with prominent non-SNV mechanisms, such as NF1 microdeletions and MECP2 duplications, are incompletely captured. The exploratory low-frequency signals did not survive multiple-testing correction, and their allele counts of two or three are sensitive to sequencing artifacts and the inclusion of related individuals in gnomAD. The EAS-NFE comparison is asymmetric in sample size and insensitive to EAS-specific enrichment below an allele frequency of about 4.5 × 10^−5^; negative findings below this floor do not imply equal frequencies. ClinVar classifications themselves carry uncertainty, and no independent ACMG/AMP re-curation was performed. We did not compute *in silico* pathogenicity scores (for example, CADD or REVEL) for the seventeen variants, because this study addresses specifically the population-frequency layer of variant interpretation; combining frequency-based evidence with *in silico* predictors remains a direction for future re-curation of the EAS-enriched variants identified here. Despite these limitations, the core findings are robust: nucleotide-resolved analysis recovers population-specific signals concentrated in recessive epilepsy genes, and rsID-level workflows systematically inflate such signals.

## 5 Conclusion

ClinVar-reported P/LP variants in epilepsy-associated genes show substantial population-specific frequency differences between EAS and NFE populations. Nine significantly enriched variants and eight additional exploratory low-frequency signals were identified, with thirteen of seventeen residing in seven autosomal recessive genes. The rsID-level workflow inflated these signals by 75.0%, and nucleotide-resolved matching should become a standard step in ClinVar-gnomAD integration. Ancestry-matched interpretation and population-aware carrier frequency estimation are actionable priorities for equitable epilepsy genetic testing across populations.

## Data Availability

The datasets presented in this study can be found in online repositories. The names of the repository/repositories and accession number(s) can be found in the article/Supplementary Material.

## Supplementary material

SUPPLEMENTARY TABLE S1 Full gene list (199 genes) with inheritance mode, phenotype, functional classification, and inclusion source.

SUPPLEMENTARY TABLE S2 Complete list of the seventeen EAS-enriched P/LP variants with allele frequencies across gnomAD v4.1.1 populations (EAS, NFE, SAS, AFR, AMR), RefSeq transcripts, molecular consequence, odds ratios, and ClinVar review status (stars).

SUPPLEMENTARY TABLE S3 The 45 rsID-level false positives with the ClinVar P/LP allele and the gnomAD most common allele for each position.

SUPPLEMENTARY TABLE S4 Full comparison of the rsID-level and nucleotide-resolved candidate sets.

SUPPLEMENTARY FIGURE S1 Flowchart of the nucleotide-resolved matching procedure. Of 9,847 unique ClinVar P/LP SNV rsIDs, 2,160 had gnomAD v4.1.1 data and 805 yielded an informative nucleotide-resolved match.

SUPPLEMENTARY FIGURE S2 Detectability of EAS-enriched variants (minimum EAS allele count required to reach a given significance threshold). The y-axis shows the negative log10 of the two-sided Fisher exact p value as a function of the EAS allele count, for variants with zero, one, or three NFE alleles. Horizontal lines mark p = 0.05 and p = 0.01. An NFE-absent variant requires at least two EAS alleles to reach p < 0.01.
